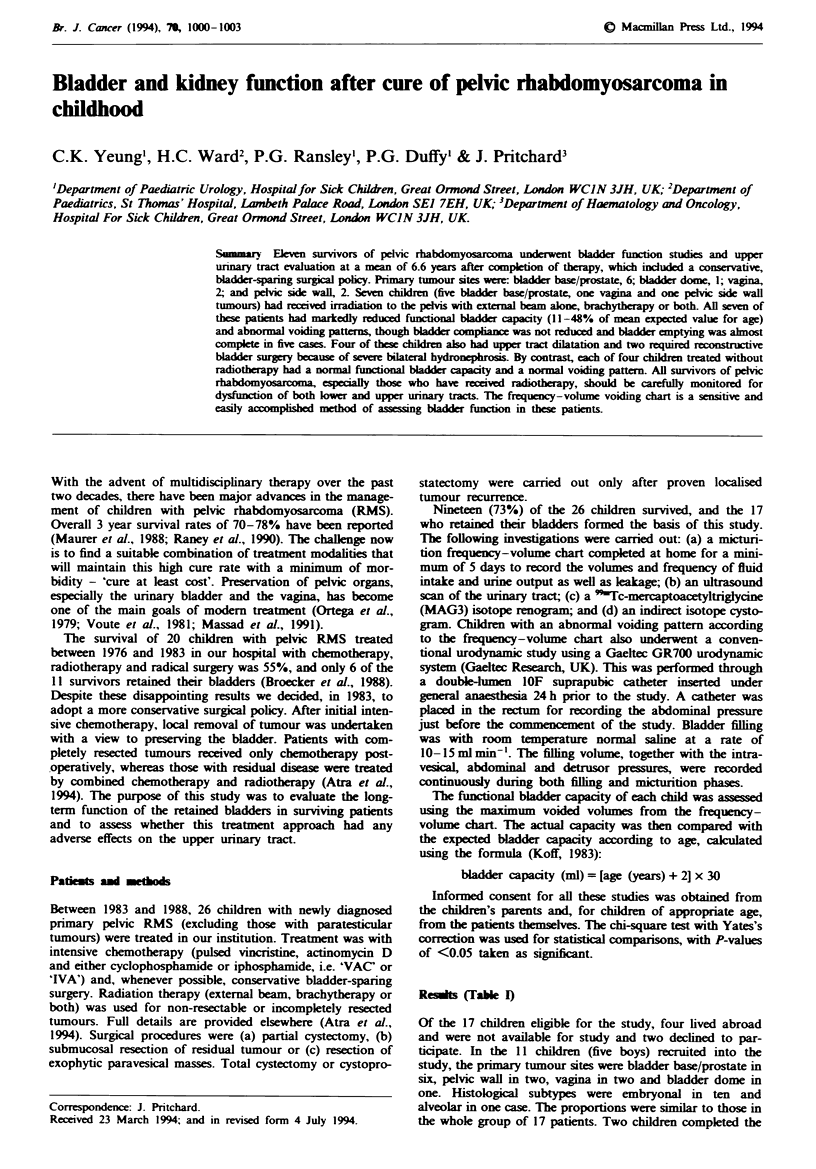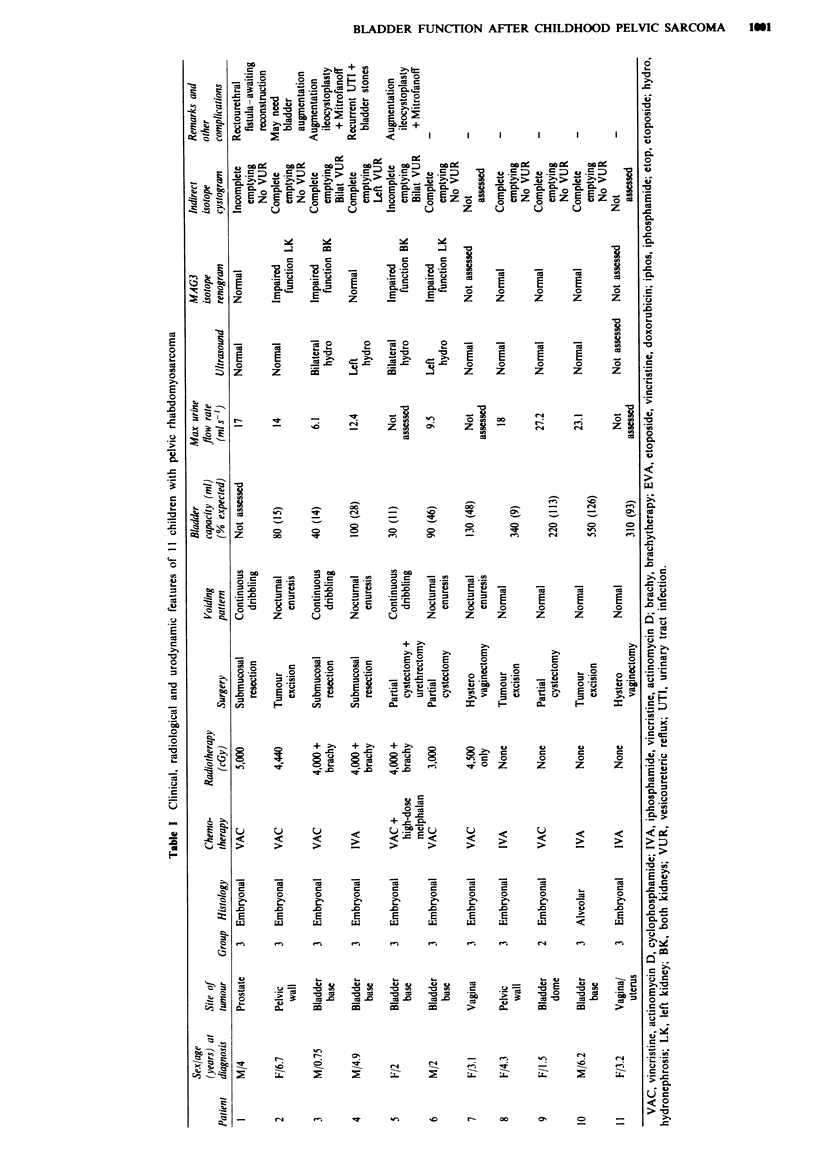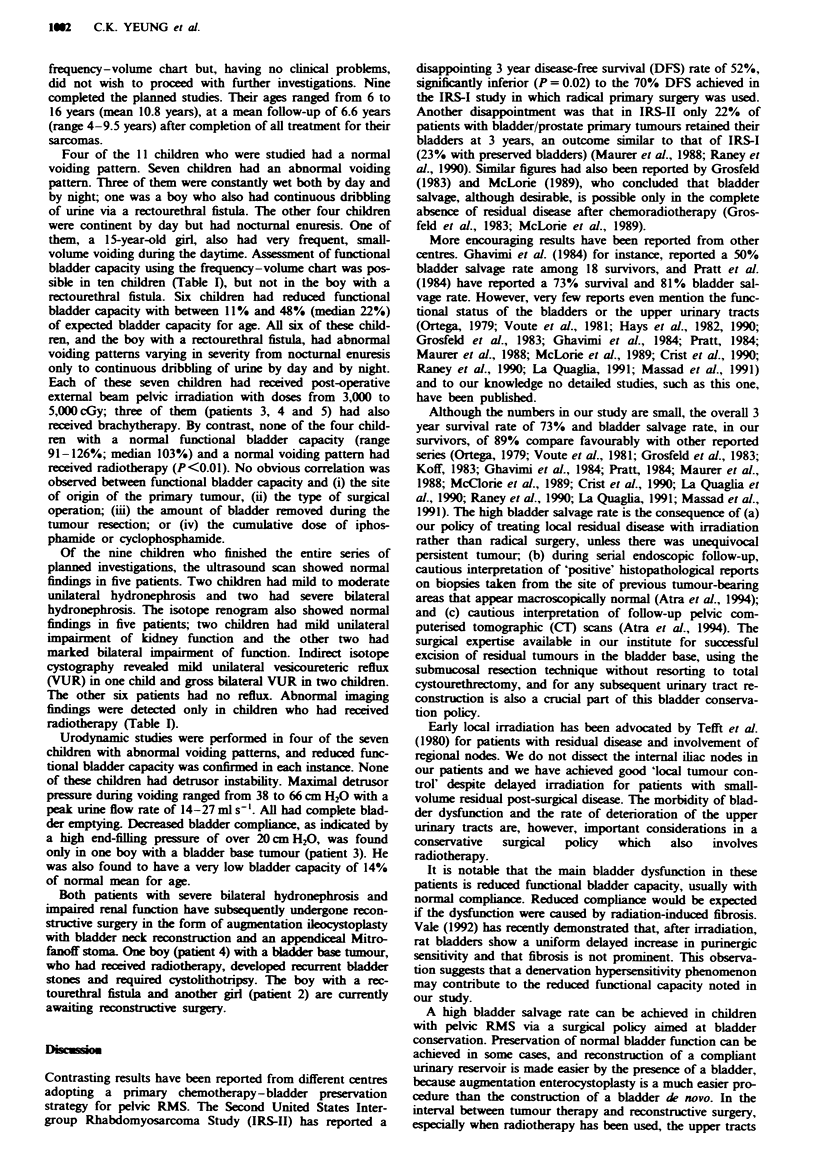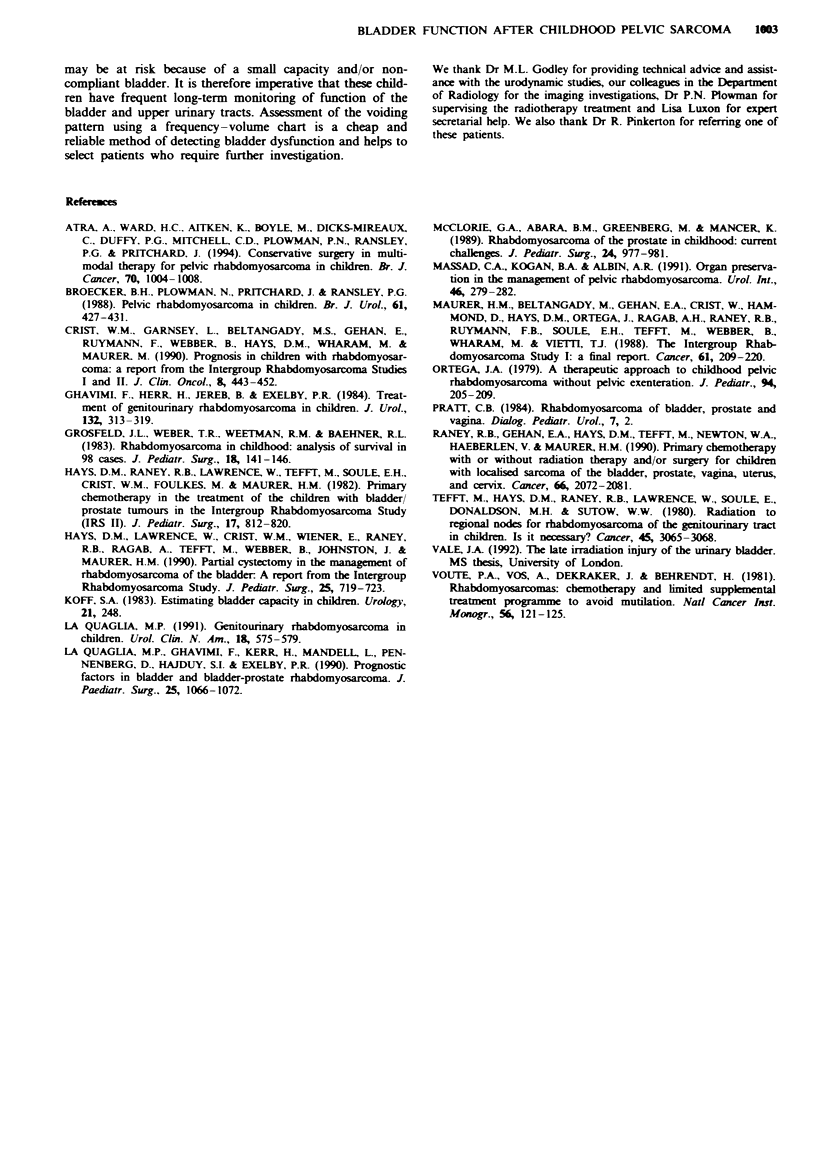# Bladder and kidney function after cure of pelvic rhabdomyosarcoma in childhood.

**DOI:** 10.1038/bjc.1994.437

**Published:** 1994-11

**Authors:** C. K. Yeung, H. C. Ward, P. G. Ransley, P. G. Duffy, J. Pritchard

**Affiliations:** Department of Paediatric Urology, Hospital for Sick Children, London, UK.

## Abstract

Eleven survivors of pelvic rhabdomyosarcoma underwent bladder function studies and upper urinary tract evaluation at a mean of 6.6 years after completion of therapy, which included a conservative, bladder-sparing surgical policy. Primary tumour sites were: bladder base/prostate, 6; bladder dome, 1; vagina, 2; and pelvic side wall, 2. Seven children (five bladder base/prostate, one vagina and one pelvic side wall tumours) had received irradiation to the pelvis with external beam alone, brachytherapy or both. All seven of these patients had markedly reduced functional bladder capacity (11-48% of mean expected value for age) and abnormal voiding patterns, though bladder compliance was not reduced and bladder emptying was almost complete in five cases. Four of these children also had upper tract dilatation and two required reconstructive bladder surgery because of severe bilateral hydronephrosis. By contrast, each of four children treated without radiotherapy had a normal functional bladder capacity and a normal voiding pattern. all survivors of pelvic rhabdomyosarcoma, especially those who have received radiotherapy, should be carefully monitored for dysfunction of both lower and upper urinary tracts. The frequency-volume voiding chart is a sensitive and easily accomplished method of assessing bladder function in these patients.


Br. J. Cancer (1994), 70, 1000-1003 © Macmillan Press Ltd., 1994

### Bladder and kidney function after cure of pelvic rhabdomyosarcoma in childhood

C.K. Yeung[1], H.C. Ward[2], P.G. Ransley[1], P.G. Duffy[1] & J. Pritchard[3]

[1]Department of Paediatric Urology, Hospital for Sick Children, Great Ormond Street, London WC1N 3JH, UK; [2]Department of Paediatrics, St Thomas' Hospital, Lambeth Palace Road, London SE1 7EH, UK; [3]Department of Haematology and Oncology, Hospital For Sick Children, Great Ormond Street, London WC1N 3JH, UK.

**Summary** Eleven survivors of pelvic rhabdomyosarcoma underwent bladder function studies and upper urinary tract evaluation at a mean of 6.6 years after completion of therapy, which included a conservative, bladder-sparing surgical policy. Primary tumour sites were: bladder base/prostate, 6; bladder dome, 1; vagina, 2; and pelvic side wall, 2. Seven children (five bladder base/prostate, one vagina and one pelvic side wall tumours) had received irradiation to the pelvis with external beam alone, brachytherapy or both. All seven of these patients had markedly reduced functional bladder capacity (11-48% of mean expected value for age) and abnormal voiding patterns, though bladder compliance was not reduced and bladder emptying was almost complete in five cases. Four of these children also had upper tract dilatation and two required reconstructive bladder surgery because of severe bilateral hydronephrosis. By contrast, each of four children treated without radiotherapy had a normal functional bladder capacity and a normal voiding pattern. All survivors of pelvic rhabdomyosarcoma, especially those who have received radiotherapy, should be carefully monitored for dysfunction of both lower and upper urinary tracts. The frequency-volume voiding chart is a sensitive and easily accomplished method of assessing bladder function in these patients.

With the advent of multidisciplinary therapy over the past two decades, there have been major advances in the management of children with pelvic rhabdomyosarcoma (RMS). Overall 3 year survival rates of 70-78% have been reported (Maurer *et al.*, 1988; Raney *et al.*, 1990). The challenge now is to find a suitable combination of treatment modalities that will maintain this high cure rate with a minimum of morbidity - 'cure at least cost'. Preservation of pelvic organs, especially the urinary bladder and the vagina, has become one of the main goals of modern treatment (Ortega *et al.*, 1979; Voute *et al.*, 1981; Massad *et al.*, 1991).

The survival of 20 children with pelvic RMS treated between 1976 and 1983 in our hospital with chemotherapy, radiotherapy and radical surgery was 55%, and only 6 of the 11 survivors retained their bladders (Broecker *et al.*, 1988). Despite these disappointing results we decided, in 1983, to adopt a more conservative surgical policy. After initial intensive chemotherapy, local removal of tumour was undertaken with a view to preserving the bladder. Patients with completely resected tumours received only chemotherapy postoperatively, whereas those with residual disease were treated by combined chemotherapy and radiotherapy (Atra *et al.*, 1994). The purpose of this study was to evaluate the long-term function of the retained bladders in surviving patients and to assess whether this treatment approach had any adverse effects on the upper urinary tract.

### Patients and methods

Between 1983 and 1988, 26 children with newly diagnosed primary pelvic RMS (excluding those with paratesticular tumours) were treated in our institution. Treatment was with intensive chemotherapy (pulsed vincristine, actinomycin D and either cyclophosphamide or iphosphamide, i.e. 'VAC' or 'IVA') and, whenever possible, conservative bladder-sparing surgery. Radiation therapy (external beam, brachytherapy or both) was used for non-resectable or incompletely resected tumours. Full details are provided elsewhere (Atra *et al.*, 1994). Surgical procedures were (a) partial cystectomy, (b) submucosal resection of residual tumour or (c) resection of exophytic paravesical masses. Total cystectomy or cystoprostatectomy were carried out only after proven localised tumour recurrence.

Nineteen (73%) of the 26 children survived, and the 17 who retained their bladders formed the basis of this study. The following investigations were carried out: (a) a micturition frequency-volume chart completed at home for a minimum of 5 days to record the volumes and frequency of fluid intake and urine output as well as leakage; (b) an ultrasound scan of the urinary tract; (c) a $^{99m}$Tc-mercaptoacetyltriglycine (MAG3) isotope renogram; and (d) an indirect isotope cystogram. Children with an abnormal voiding pattern according to the frequency-volume chart also underwent a conventional urodynamic study using a Gaeltec GR700 urodynamic system (Gaeltec Research, UK). This was performed through a double-lumen 10F suprapubic catheter inserted under general anaesthesia 24 h prior to the study. A catheter was placed in the rectum for recording the abdominal pressure just before the commencement of the study. Bladder filling was with room temperature normal saline at a rate of 10-15 ml min$^{-1}$. The filling volume, together with the intravesical, abdominal and detrusor pressures, were recorded continuously during both filling and micturition phases.

The functional bladder capacity of each child was assessed using the maximum voided volumes from the frequency-volume chart. The actual capacity was then compared with the expected bladder capacity according to age, calculated using the formula (Koff, 1983):

$$\text{bladder capacity (ml)} = [\text{age (years)} + 2] \times 30$$

Informed consent for all these studies was obtained from the children's parents and, for children of appropriate age, from the patients themselves. The chi-square test with Yates's correction was used for statistical comparisons, with $P$-values of $<0.05$ taken as significant.

### Results (Table I)

Of the 17 children eligible for the study, four lived abroad and were not available for study and two declined to participate. In the 11 children (five boys) recruited into the study, the primary tumour sites were bladder base/prostate in six, pelvic wall in two, vagina in two and bladder dome in one. Histological subtypes were embryonal in ten and alveolar in one case. The proportions were similar to those in the whole group of 17 patients. Two children completed the

Correspondence: J. Pritchard.
Received 23 March 1994; and in revised form 4 July 1994.

**Table I** Clinical, radiological and urodynamic features of 11 children with pelvic rhabdomyosarcoma

| *Patient* | *Sex/age (years) at diagnosis* | *Site of tumour* | *Group* | *Histology* | *Chemo-therapy* | *Radiotherapy (cGy)* | *Surgery* | *Voiding pattern* | *Bladder capacity (ml) (% expected)* | *Max urine flow rate* ($ml\,s^{-1}$) | *Ultrasound* | *MAG3 isotope renogram* | *Indirect isotope cystogram* | *Remarks and other complications* |
|---|---|---|---|---|---|---|---|---|---|---|---|---|---|---|
| 1 | M/4 | Prostate | 3 | Embryonal | VAC | 5,000 | Submucosal resection | Continuous dribbling | Not assessed | 17 | Normal | Normal | Incomplete emptying No VUR | Rectourethral fistula-awaiting reconstruction |
| 2 | F/6.7 | Pelvic wall | 3 | Embryonal | VAC | 4,440 | Tumour excision | Nocturnal enuresis | 80 (15) | 14 | Normal | Impaired function LK | Complete emptying No VUR | May need bladder augmentation |
| 3 | M/0.75 | Bladder base | 3 | Embryonal | VAC | 4,000 + brachy | Submucosal resection | Continuous dribbling | 40 (14) | 6.1 | Bilateral hydro | Impaired function BK | Complete emptying Bilat VUR | Augmentation ileocystoplasty + Mitrofanoff |
| 4 | M/4.9 | Bladder base | 3 | Embryonal | IVA | 4,000 + brachy | Submucosal resection | Nocturnal enuresis | 100 (28) | 12.4 | Left hydro | Normal | Complete emptying Left VUR | Recurrent UTI + bladder stones |
| 5 | F/2 | Bladder base | 3 | Embryonal | VAC + high-dose melphalan | 4,000 + brachy | Partial cystectomy + urethrectomy | Continuous dribbling | 30 (11) | Not assessed | Bilateral hydro | Impaired function BK | Incomplete emptying Bilat VUR | Augmentation ileocystoplasty + Mitrofanoff |
| 6 | M/2 | Bladder base | 3 | Embryonal | VAC | 3,000 | Partial cystectomy | Nocturnal enuresis | 90 (46) | 9.5 | Left hydro | Impaired function LK | Complete emptying No VUR | – |
| 7 | F/3.1 | Vagina | 3 | Embryonal | VAC | 4,500 only | Hystero vaginectomy | Nocturnal enuresis | 130 (48) | Not assessed | Normal | Not assessed | Not assessed | – |
| 8 | F/4.3 | Pelvic wall | 3 | Embryonal | IVA | None | Tumour excision | Normal | 340 (9) | 18 | Normal | Normal | Complete emptying No VUR | – |
| 9 | F/1.5 | Bladder dome | 2 | Embryonal | VAC | None | Partial cystectomy | Normal | 220 (113) | 27.2 | Normal | Normal | Complete emptying No VUR | – |
| 10 | M/6.2 | Bladder base | 3 | Alveolar | IVA | None | Tumour excision | Normal | 550 (126) | 23.1 | Normal | Normal | Complete emptying No VUR | – |
| 11 | F/3.2 | Vagina/ uterus | 3 | Embryonal | IVA | None | Hystero vaginectomy | Normal | 310 (93) | Not assessed | Not assessed | Not assessed | Not assessed | – |

VAC, vincristine, actinomycin D, cyclophosphamide; IVA, iphosphamide, vincristine, actinomycin D; brachy, brachytherapy; EVA, etoposide, vincristine, doxorubicin; iphos, iphosphamide; etop, etoposide; hydro, hydronephrosis; LK, left kidney; BK, both kidneys; VUR, vesicoureteric reflux; UTI, urinary tract infection.

frequency–volume chart but, having no clinical problems, did not wish to proceed with further investigations. Nine completed the planned studies. Their ages ranged from 6 to 16 years (mean 10.8 years), at a mean follow-up of 6.6 years (range 4–9.5 years) after completion of all treatment for their sarcomas.

Four of the 11 children who were studied had a normal voiding pattern. Seven children had an abnormal voiding pattern. Three of them were constantly wet both by day and by night; one was a boy who also had continuous dribbling of urine via a rectourethral fistula. The other four children were continent by day but had nocturnal enuresis. One of them, a 15-year-old girl, also had very frequent, small-volume voiding during the daytime. Assessment of functional bladder capacity using the frequency–volume chart was possible in ten children (Table I), but not in the boy with a rectourethral fistula. Six children had reduced functional bladder capacity with between 11% and 48% (median 22%) of expected bladder capacity for age. All six of these children, and the boy with a rectourethral fistula, had abnormal voiding patterns varying in severity from nocturnal enuresis only to continuous dribbling of urine by day and by night. Each of these seven children had received post-operative external beam pelvic irradiation with doses from 3,000 to 5,000 cGy; three of them (patients 3, 4 and 5) had also received brachytherapy. By contrast, none of the four children with a normal functional bladder capacity (range 91–126%; median 103%) and a normal voiding pattern had received radiotherapy ($P<0.01$). No obvious correlation was observed between functional bladder capacity and (i) the site of origin of the primary tumour, (ii) the type of surgical operation; (iii) the amount of bladder removed during the tumour resection; or (iv) the cumulative dose of iphosphamide or cyclophosphamide.

Of the nine children who finished the entire series of planned investigations, the ultrasound scan showed normal findings in five patients. Two children had mild to moderate unilateral hydronephrosis and two had severe bilateral hydronephrosis. The isotope renogram also showed normal findings in five patients; two children had mild unilateral impairment of kidney function and the other two had marked bilateral impairment of function. Indirect isotope cystography revealed mild unilateral vesicoureteric reflux (VUR) in one child and gross bilateral VUR in two children. The other six patients had no reflux. Abnormal imaging findings were detected only in children who had received radiotherapy (Table I).

Urodynamic studies were performed in four of the seven children with abnormal voiding patterns, and reduced functional bladder capacity was confirmed in each instance. None of these children had detrusor instability. Maximal detrusor pressure during voiding ranged from 38 to 66 cm $H_2O$ with a peak urine flow rate of 14–27 ml $s^{-1}$. All had complete bladder emptying. Decreased bladder compliance, as indicated by a high end-filling pressure of over 20 cm $H_2O$, was found only in one boy with a bladder base tumour (patient 3). He was also found to have a very low bladder capacity of 14% of normal mean for age.

Both patients with severe bilateral hydronephrosis and impaired renal function have subsequently undergone reconstructive surgery in the form of augmentation ileocystoplasty with bladder neck reconstruction and an appendiceal Mitrofanoff stoma. One boy (patient 4) with a bladder base tumour, who had received radiotherapy, developed recurrent bladder stones and required cystolithotripsy. The boy with a rectourethral fistula and another girl (patient 2) are currently awaiting reconstructive surgery.

### Discussion

Contrasting results have been reported from different centres adopting a primary chemotherapy–bladder preservation strategy for pelvic RMS. The Second United States Intergroup Rhabdomyosarcoma Study (IRS-II) has reported a disappointing 3 year disease-free survival (DFS) rate of 52%, significantly inferior ($P = 0.02$) to the 70% DFS achieved in the IRS-I study in which radical primary surgery was used. Another disappointment was that in IRS-II only 22% of patients with bladder/prostate primary tumours retained their bladders at 3 years, an outcome similar to that of IRS-I (23% with preserved bladders) (Maurer *et al.*, 1988; Raney *et al.*, 1990). Similar figures had also been reported by Grosfeld (1983) and McLorie (1989), who concluded that bladder salvage, although desirable, is possible only in the complete absence of residual disease after chemoradiotherapy (Grosfeld *et al.*, 1983; McLorie *et al.*, 1989).

More encouraging results have been reported from other centres. Ghavimi *et al.* (1984) for instance, reported a 50% bladder salvage rate among 18 survivors, and Pratt *et al.* (1984) have reported a 73% survival and 81% bladder salvage rate. However, very few reports even mention the functional status of the bladders or the upper urinary tracts (Ortega, 1979; Voute *et al.*, 1981; Hays *et al.*, 1982, 1990; Grosfeld *et al.*, 1983; Ghavimi *et al.*, 1984; Pratt, 1984; Maurer *et al.*, 1988; McLorie *et al.*, 1989; Crist *et al.*, 1990; Raney *et al.*, 1990; La Quaglia, 1991; Massad *et al.*, 1991) and to our knowledge no detailed studies, such as this one, have been published.

Although the numbers in our study are small, the overall 3 year survival rate of 73% and bladder salvage rate, in our survivors, of 89% compare favourably with other reported series (Ortega, 1979; Voute *et al.*, 1981; Grosfeld *et al.*, 1983; Koff, 1983; Ghavimi *et al.*, 1984; Pratt, 1984; Maurer *et al.*, 1988; McClorie *et al.*, 1989; Crist *et al.*, 1990; La Quaglia *et al.*, 1990; Raney *et al.*, 1990; La Quaglia, 1991; Massad *et al.*, 1991). The high bladder salvage rate is the consequence of (a) our policy of treating local residual disease with irradiation rather than radical surgery, unless there was unequivocal persistent tumour; (b) during serial endoscopic follow-up, cautious interpretation of 'positive' histopathological reports on biopsies taken from the site of previous tumour-bearing areas that appear macroscopically normal (Atra *et al.*, 1994); and (c) cautious interpretation of follow-up pelvic computerised tomographic (CT) scans (Atra *et al.*, 1994). The surgical expertise available in our institute for successful excision of residual tumours in the bladder base, using the submucosal resection technique without resorting to total cystourethrectomy, and for any subsequent urinary tract reconstruction is also a crucial part of this bladder conservation policy.

Early local irradiation has been advocated by Tefft *et al.* (1980) for patients with residual disease and involvement of regional nodes. We do not dissect the internal iliac nodes in our patients and we have achieved good 'local tumour control' despite delayed irradiation for patients with small-volume residual post-surgical disease. The morbidity of bladder dysfunction and the rate of deterioration of the upper urinary tracts are, however, important considerations in a conservative surgical policy which also involves radiotherapy.

It is notable that the main bladder dysfunction in these patients is reduced functional bladder capacity, usually with normal compliance. Reduced compliance would be expected if the dysfunction were caused by radiation-induced fibrosis. Vale (1992) has recently demonstrated that, after irradiation, rat bladders show a uniform delayed increase in purinergic sensitivity and that fibrosis is not prominent. This observation suggests that a denervation hypersensitivity phenomenon may contribute to the reduced functional capacity noted in our study.

A high bladder salvage rate can be achieved in children with pelvic RMS via a surgical policy aimed at bladder conservation. Preservation of normal bladder function can be achieved in some cases, and reconstruction of a compliant urinary reservoir is made easier by the presence of a bladder, because augmentation enterocystoplasty is a much easier procedure than the construction of a bladder *de novo*. In the interval between tumour therapy and reconstructive surgery, especially when radiotherapy has been used, the upper tracts may be at risk because of a small capacity and/or non-compliant bladder. It is therefore imperative that these children have frequent long-term monitoring of function of the bladder and upper urinary tracts. Assessment of the voiding pattern using a frequency-volume chart is a cheap and reliable method of detecting bladder dysfunction and helps to select patients who require further investigation.

We thank Dr M.L. Godley for providing technical advice and assistance with the urodynamic studies, our colleagues in the Department of Radiology for the imaging investigations, Dr P.N. Plowman for supervising the radiotherapy treatment and Lisa Luxon for expert secretarial help. We also thank Dr R. Pinkerton for referring one of these patients.

### References

ATRA, A., WARD, H.C., AITKEN, K., BOYLE, M., DICKS-MIREAUX, C., DUFFY, P.G., MITCHELL, C.D., PLOWMAN, P.N., RANSLEY, P.G. & PRITCHARD, J. (1994). Conservative surgery in multimodal therapy for pelvic rhabdomyosarcoma in children. *Br. J. Cancer*, **70**, 1004-1008.

BROECKER, B.H., PLOWMAN, N., PRITCHARD, J. & RANSLEY, P.G. (1988). Pelvic rhabdomyosarcoma in children. *Br. J. Urol.*, **61**, 427-431.

CRIST, W.M., GARNSEY, L., BELTANGADY, M.S., GEHAN, E., RUYMANN, F., WEBBER, B., HAYS, D.M., WHARAM, M. & MAURER, M. (1990). Prognosis in children with rhabdomyosarcoma: a report from the Intergroup Rhabdomyosarcoma Studies I and II. *J. Clin. Oncol.*, **8**, 443-452.

GHAVIMI, F., HERR, H., JEREB, B. & EXELBY, P.R. (1984). Treatment of genitourinary rhabdomyosarcoma in children. *J. Urol.*, **132**, 313-319.

GROSFELD, J.L., WEBER, T.R., WEETMAN, R.M. & BAEHNER, R.L. (1983). Rhabdomyosarcoma in childhood: analysis of survival in 98 cases. *J. Pediatr. Surg.*, **18**, 141-146.

HAYS, D.M., RANEY, R.B., LAWRENCE, W., TEFFT, M., SOULE, E.H., CRIST, W.M., FOULKES, M. & MAURER, H.M. (1982). Primary chemotherapy in the treatment of the children with bladder/prostate tumours in the Intergroup Rhabdomyosarcoma Study (IRS II). *J. Pediatr. Surg.*, **17**, 812-820.

HAYS, D.M., LAWRENCE, W., CRIST, W.M., WIENER, E., RANEY, R.B., RAGAB, A., TEFFT, M., WEBBER, B., JOHNSTON, J. & MAURER, H.M. (1990). Partial cystectomy in the management of rhabdomyosarcoma of the bladder: A report from the Intergroup Rhabdomyosarcoma Study. *J. Pediatr. Surg.*, **25**, 719-723.

KOFF, S.A. (1983). Estimating bladder capacity in children. *Urology*, **21**, 248.

LA QUAGLIA, M.P. (1991). Genitourinary rhabdomyosarcoma in children. *Urol. Clin. N. Am.*, **18**, 575-579.

LA QUAGLIA, M.P., GHAVIMI, F., KERR, H., MANDELL, L., PENNENBERG, D., HAJDUY, S.I. & EXELBY, P.R. (1990). Prognostic factors in bladder and bladder-prostate rhabdomyosarcoma. *J. Paediatr. Surg.*, **25**, 1066-1072.

McCLORIE, G.A., ABARA, B.M., GREENBERG, M. & MANCER, K. (1989). Rhabdomyosarcoma of the prostate in childhood: current challenges. *J. Pediatr. Surg.*, **24**, 977-981.

MASSAD, C.A., KOGAN, B.A. & ALBIN, A.R. (1991). Organ preservation in the management of pelvic rhabdomyosarcoma. *Urol. Int.*, **46**, 279-282.

MAURER, H.M., BELTANGADY, M., GEHAN, E.A., CRIST, W., HAMMOND, D., HAYS, D.M., ORTEGA, J., RAGAB, A.H., RANEY, R.B., RUYMANN, F.B., SOULE, E.H., TEFFT, M., WEBBER, B., WHARAM, M. & VIETTI, T.J. (1988). The Intergroup Rhabdomyosarcoma Study I: a final report. *Cancer*, **61**, 209-220.

ORTEGA, J.A. (1979). A therapeutic approach to childhood pelvic rhabdomyosarcoma without pelvic exenteration. *J. Pediatr.*, **94**, 205-209.

PRATT, C.B. (1984). Rhabdomyosarcoma of bladder, prostate and vagina. *Dialog. Pediatr. Urol.*, **7**, 2.

RANEY, R.B., GEHAN, E.A., HAYS, D.M., TEFFT, M., NEWTON, W.A., HAEBERLEN, V. & MAURER, H.M. (1990). Primary chemotherapy with or without radiation therapy and/or surgery for children with localised sarcoma of the bladder, prostate, vagina, uterus, and cervix. *Cancer*, **66**, 2072-2081.

TEFFT, M., HAYS, D.M., RANEY, R.B., LAWRENCE, W., SOULE, E., DONALDSON, M.H. & SUTOW, W.W. (1980). Radiation to regional nodes for rhabdomyosarcoma of the genitourinary tract in children. Is it necessary? *Cancer*, **45**, 3065-3068.

VALE, J.A. (1992). The late irradiation injury of the urinary bladder. MS thesis, University of London.

VOUTE, P.A., VOS, A., DEKRAKER, J. & BEHRENDT, H. (1981). Rhabdomyosarcomas: chemotherapy and limited supplemental treatment programme to avoid mutilation. *Natl Cancer Inst. Monogr.*, **56**, 121-125.